# Incorporating High-Risk Individuals Beyond Smoking History Into Lung Cancer Screening in Hong Kong: A Cost-Effectiveness Study

**DOI:** 10.1016/j.jtocrr.2025.100860

**Published:** 2025-06-13

**Authors:** Herbert Ho-fung Loong, Xuanqi Pan, Carlos K.H. Wong, Chao-Hua Chiu, Szu-Chun Yang, Matthew Shing Hin Chung, Molly Siu Ching Li, Lisa de Jong, Harry Groen, Maarten J. Postma, Pan-Chyr Yang

**Affiliations:** a Department of Clinical Oncology, Faculty of Medicine, The Chinese University of Hong Kong, Hong Kong SAR, People's Republic of China; b Institute for Diagnostic Accuracy, Groningen, The Netherlands; c Unit of Global Health, Faculty of Medical Sciences, University of Groningen, Groningen, The Netherlands; d Department of Pharmacology and Pharmacy, LKS Faculty of Medicine, University of Hong Kong, Hong Kong SAR, People's Republic of China; e Department of Family Medicine and Primary Care, School of Clinical Medicine, LKS Faculty of Medicine, University of Hong Kong, Hong Kong SAR, People's Republic of China; f Laboratory of Data Discovery for Health (D^2^4H), Hong Kong SAR, People's Republic of China; g Taipei Cancer Center and Taipei Medical University Hospital, Taipei, Taiwan; h Department of Internal Medicine, National Cheng Kung University Hospital, College of Medicine, National Cheng Kung University, Tainan, Taiwan; i Department of Medicine, School of Clinical Medicine, LKS Faculty of Medicine, University of Hong Kong, Hong Kong SAR, People's Republic of China; j Faculty of Medical Sciences, University of Groningen, Groningen, The Netherlands; k Department of Economics, Econometrics & Finance, University of Groningen, Faculty of Economics and Business, Groningen, The Netherlands; l Center of Excellence for Pharmaceutical Care Innovation, Universitas Padjadjaran, Bandung, Indonesia; m Division of Pharmacology and Therapy, Faculty of Medicine Universitas Airlangga, Surabaya, Indonesia; n Department of Internal Medicine, National Taiwan University Hospital and National Taiwan University College of Medicine, Taipei, Taiwan; o Department of Infectious Disease Epidemiology and Dynamics, Faculty of Epidemiology and Population Health, London School of Hygiene and Tropical Medicine, London, United Kingdom

**Keywords:** Lung cancer, Lung cancer screening, LDCT, High-risk individuals, Smoking history

## Abstract

**Introduction:**

Lung cancer (LC) accounts for 26.4% of all cancer deaths in Hong Kong (HK). Lung cancer screening (LCS) with low-dose computed tomography (LDCT) can reduce LC mortality. The cost-effectiveness of LDCT screening in high-risk individuals on the basis of smoking history has previously been investigated. However, nearly half of patients with LC in HK never smoke, indicating a different LC epidemiology compared with Western countries, where most LC cases are associated with smoking. We conducted a cost-effectiveness analysis for LCS, utilizing local data and expanding the target population to include we not only high-risk individuals identified on the basis of smoking history but also those identified through other risk factors.

**Methods:**

A decision tree combined with a state-transition Markov model was developed to simulate identification, diagnosis, and treatments for high-risk individuals, from a health care provider perspective. The selection criteria and screening effectiveness for high-risk individuals on the basis of smoking history were obtained from the Dutch-Belgian Lung Cancer Screening Study, targeting heavy smokers aged 50 to 74 years; whereas the Taiwan Lung Cancer Screening in Never-Smoker Trial was used to model high-risk individuals on the basis of factors other than smoking history. Local LC survival and cost data were used to populate the model. The willingness-to-pay threshold used in the study was US$24,302 to US$40,202 per quality-adjusted life-year (QALY).

**Results:**

Screening led to additional early LC detected, and LC mortality reduction, compared with no screening. Over a lifetime horizon, the incremental cost-effectiveness ratio for high-risk individuals on the basis of smoking history was US$14,122 per QALY. The incremental cost-effectiveness ratio for high-risk individuals on the basis of factors other than smoking history was lower at US$9610 per QALY.

**Conclusion:**

LCS with LDCT can be considered cost-effective in HK for high-risk individuals on the basis of smoking history and factors other than smoking history, contributing to the health benefits of the population. Our findings support a population-based LCS for all high-risk individuals identified through criteria beyond smoking history.

## Introduction

Lung cancer is the most common cancer type and the leading cause of cancer-related death worldwide.^1^ In Hong Kong (HK), lung cancer accounted for 15.5% of all new cancer cases (5978 cases) in 2021, and 26.4% of all cancer deaths (3782) in 2022.^2^ Moreover, there is a significant trend of increasing incidence and mortality in lung cancer, primarily attributed to an aging and growing population.^3^ Early detection of lung cancer is essential to reduce this heavy disease burden, as lung cancer survival is significantly better for patients with lung cancer detected at an earlier stage, given the availability of curative-intent treatments.^4^ Studies reported that lung cancer screening (LCS) with low-dose computed tomography (LDCT) can detect lung cancer at early stages and reduce mortality. The National Lung Screening Trial (NLST) is a prospective randomized controlled clinical study that reported a 20% mortality reduction in patients randomized to LDCT when compared with the conventional chest radiography screening among a high-risk smoking population in the United States.^5^ The Dutch-Belgian Lung Cancer Screening Study (Dutch acronym: NELSON) reported that LCS with volume computed tomography (CT) could reduce mortality by 24% to 33% in a high-risk population who are currently smoking or have a recent smoking history.^6^

On the basis of these studies, authoritative medical organizations have issued recommendations and launched screening guidelines for LCS in high-risk groups with LDCT.^7^, ^8^, ^9^, ^10^ Generally, age and smoking history with regard to chronicity and amount of cigarette exposure are the main criteria to define high-risk groups. However, other risk factors, such as air pollution, passive smoking, cooking without air ventilation, and family history, are suspected to contribute to lung cancer development.^11^^,^^12^ This holds especially in Asia. Whereas previous studies have reported the proportion of female patients with lung cancer having no smoking history being in the range of 50% in Europe and 10% in the United States,^13^ in contrast, studies from the People's Republic of China and Japan have reported over 90% and around 75% of female patients with lung cancer having no smoking history, respectively.^14^ This indicates that lung cancer in Asia has a different epidemiologic profile compared with Western countries.^15^^,^^16^ To reflect this regional distinction, The Taiwan Lung Cancer Screening in Never-Smoker Trial (TALENT) focused primarily on high-risk individuals identified by factors other than smoking history.^17^

Though the cost-effectiveness of LDCT screening targeting populations with significant histories of smoking has been investigated in various countries, the cost-effectiveness of LCS for a mixed cohort, consisting of high-risk individuals on the basis of smoking history and on factors other than smoking history, has yet to be explored.^18^, ^19^, ^20^, ^21^ This modeling study aimed to investigate whether LCS is cost-effective when incorporating such a mixed screening cohort. Using known epidemiology, investigations and treatment costs, and estimated clinical benefits for patients with lung cancer in HK, we aim to provide recommendations for LCS implementation.

## Methods

A cost-effectiveness analysis was conducted, using a decision tree and a cohort state-transition Markov model to simulate the identification, diagnosis, and treatments, for individuals at high risk of developing lung cancer. The model type and structure rationale were elucidated comprehensively in a previous study.^22^ Eligible smoker participants were assumed to undergo annual volume-based LDCT screening on the basis of the NELSON study, with the inclusion criteria being age (50 -74 y), and a history of smoking. The latter is defined as “having smoked more than 15 cigarettes per day for over 25 years or more than 10 cigarettes per day for over 30 years and being a current smoker or having quit smoking within the past 10 years”.^6^ For the nonsmoking population, the eligibility to receive LDCT screening was on the basis of the inclusion criteria of the TALENT study, namely: (1) age (55–75 y); (2) never-smokers or had quit for more than 15 years; and (3) having one of the following risks: family lung cancer history, environmental tobacco smoking history, chronic lung disease, cooking without using ventilation, or cooking index greater than or equal to110.^17^ This was compared with a situation of no screening for the same cohort. The study adopted a health care provider perspective, encompassing costs from both the public and private sectors, to accurately reflect the prevailing clinical practices in HK. This approach was justified considering that the cervical and colorectal cancer screening programs, which have already been implemented in HK, were both funded and coordinated through a public-private partnership model.^23^ Expert opinions confirmed that neither sector could be considered negligible, warranting their inclusion in the analysis.

The cost-effectiveness analysis followed the National Institute for Health and Care Excellence (NICE) technology appraisal methodology.^24^ The primary health outcomes were the quality-adjusted life years (QALYs) and life-years gained (LYG). The primary health economic outcome was the incremental cost-effectiveness ratio (ICER), calculated by dividing incremental costs by incremental effects (i.e., QALYs). A 3.5% annual discount rate was used for both health outcomes and monetary outcomes.^24^ A lifetime horizon was taken to fully capture the long-term health benefits and costs. The willingness-to-pay (WTP) threshold used in this study was US$24,302 to 40,202 per QALY, and this specific amount has also been agreed on in expert consultations.^25^

### Model Structure

A decision tree was used to simulate the screening approach on the basis of the NELSON study for high-risk individuals on the basis of smoking history, and details were outlined in a previous publication.^22^ A second decision tree was developed to simulate the screening strategy and effectiveness on the basis of the TALENT study, for high-risk individuals on the basis of factors other than smoking history (Supplementary Fig. 1).

To simulate the disease progress and treatments for patients with lung cancer by stage at diagnosis, a cohort state-transition Markov model was developed on the basis of the natural history of lung cancer. This model consists of three health states: (1) preprogression; (2) postprogression; and (3) death, with a 3-month cycle length.^22^

### Model Inputs

Model inputs consisted of the following: (1) size and characteristics of the eligible population; (2) screening outcomes (on the basis of the NELSON and TALENT studies); (3) lung cancer epidemiology; (4) survival data; (5) health utilities; and (6) costs. The model parameters used in the base-case analysis are presented in Table 1.

**Table 1:** Input Parameters for Screening Outcomes, Epidemiology and Demography, and Utilities

Parameter	Base-case Value	PSA Distribution
Screening outcomes (NELSON)		
NELSON round 1^26^		
Regular scan		
Negative	79.21%	Dirichlet
Indeterminate	19.20%	Dirichlet
Positive	1.59%	Dirichlet
Indeterminate scan		
Negative	94.57%	Dirichlet
Positive	5.43%	Dirichlet
True negative	99.93%	Dirichlet
False-negative	0.07%	Dirichlet
True positive^a^	38.67%	Dirichlet
False-positive^a^	61.33%	Dirichlet
Stage distribution		
Stage I	64.86%	Dirichlet
Stage II	9.46%	Dirichlet
Stage III	18.92%	Dirichlet
Stage IV	6.76%	Dirichlet
NELSON round 2^26^		
Regular scan		
Negative	92.17%	Dirichlet
Indeterminate	6.58%	Dirichlet
Positive	1.25%	Dirichlet
Indeterminate scan		
Negative	91.23%	Dirichlet
Positive	8.77%	Dirichlet
True negative	99.90%	Dirichlet
False-negative	0.10%	Dirichlet
True positive^a^	44.35%	Dirichlet
False-positive^a^	55.65%	Dirichlet
Stage distribution		
Stage I	75.86%	Dirichlet
Stage II	6.90%	Dirichlet
Stage III	13.79%	Dirichlet
Stage IV	3.45%	Dirichlet
Mean age NELSON study^26^	58.00	N.A.
Screening uptake rate^27^	46.5%	Beta
Adherence rate (Round 1 to Round 2)^b^	100%	Beta
Adherence rate (Round 2 onwards)^b^	100%	Beta
Screening outcomes (TALENT)^17^		
Negative	82.57%	Dirichlet
Positive	17.43%	Dirichlet
True negative	99.75%	Dirichlet
False-negative	0.25%	Dirichlet
True positive^a^	13.99%	Dirichlet
False-positive^a^	86.01%	Dirichlet
Stage distribution		
Stage 0	19.18%	Dirichlet
Stage I	77.36%	Dirichlet
Stage II	0.94%	Dirichlet
Stage III	0.94%	Dirichlet
Stage IV	1.57%	Dirichlet
Epidemiology and demography		
Total population^28^	7,333,200	Gamma
Population aged 50–74 y (NELSON)^29^	37.32%	Beta
Population aged 55–74 y (TALENT)^29^	29.46%	Beta
Male (proportion)^29^	45.60%	Beta
Female (proportion)^29^	54.40%	Beta
Daily smokers^30^	10.4%	Beta
Ex-smokers^30^	9.4%	Beta
Lung cancer incidence aged 50–74 y (NELSON)^3^	3,290	Gamma
Lung cancer incidence aged 55–75 y (TALENT)^3^	3,028	Gamma
Stage distribution (no screening)^31^		
Stage I	17.32%	Dirichlet
Stage II	4.76%	Dirichlet
Stage III	14.61%	Dirichlet
Stage IV	63.31%	Dirichlet
Overall survival (5-year survival rate) by stage at diagnosis (experts’ inputs)		
Stage I	47.95%	N.A.
Stage II	37.72%	N.A.
Stage III	15.36%	N.A.
Stage IV	4.82%	N.A.
Disease/progression-free survival (1-year disease/progression-free survival rate)^32^, ^33^, ^34^, ^35^, ^36^, ^37^		
Stage I	72.03%	N.A.
Stage II	72.03%	N.A.
Stage III	49.15%	N.A.
Stage IV	38.29%	N.A.

N.A., not applicable; PSA, probabilistic sensitivity analysis.

a The term “true positive” refers to the proportion of true positive scans among the total positive results, whereas the term “false-positive” refers to the proportion of false-positive scans among the total positive results, and the sum of “true positive” and “false-positive” equals to 100%.

b Assumptions on the basis of expert opinions.

### Eligible Population

On the basis of official census data provided by the HK Thematic Household Survey which studied the patterns of smoking in the population published in 2021, among a total population of 7.33 million, the number of current smokers aged 50 years and older with a daily consumption of more than 10 cigarettes was estimated at 133,000.^28^, ^29^, ^30^ An additional 107,910 individuals met the same criteria as ex-smokers.^28^, ^29^, ^30^ Thus, the total number of high-risk individuals eligible for LCS on the basis of smoking history was estimated at 240,910 individuals (3.29% of the population). According to the same survey, 1,962,185 individuals aged 55 to 74 years in HK were classified as not currently smoking.^30^ Drawing on estimates from an epidemiologic study, approximately 10% of this group were considered at high risk for lung cancer owing to factors unrelated to smoking history,^38^ such as family history and cumulative exposure to cooking fumes as outlined by the TALENT study.^17^ This yields an estimated 196,218 individuals eligible for LCS on the basis of risk factors other than smoking (2.68% of the population). In total, 437,128 high-risk individuals were eligible for LCS. The current study adopted a screening uptake rate of 46.5%, which was calibrated on the basis of the UK Lung Cancer Screening program.^27^ Subsequently, the total screening population was determined to comprise 203,265 individuals. For this analysis, complete adherence to LCS across all screening rounds was assumed.

### Screening Outcomes for High-Risk Individuals Based on Smoking History

For high-risk individuals on the basis of smoking history, the screening effectiveness, including lung cancer screen detectability and stage distribution, was derived from the NELSON study.^26^ This was used to inform the transition of screening individuals in the screening arm and the lung cancer stage distribution after individuals were screened positive. In the NELSON study, the baseline screening (round 1) detected relatively more patients with stage III and IV lung cancer than future screening rounds, as patients who had missed the opportunity to be detected earlier through screening were cumulated in the first screening round. Therefore, the baseline screening effectiveness in the model was on the basis of NELSON round 1 results, whereas the subsequent rounds in the model were on the basis of NELSON round 2 results. Cancers occurring during screening intervals were also considered in the model and followed the clinical presentation diagnoses per stage. The screening was modeled for 10 rounds, given that the NELSON study was followed up for 10 years, and illustrating impact over 10 years provides policy-relevant insights for reimbursement and resource allocation.

### Screening Outcomes for High-Risk Individuals Based on Factors Other Than Smoking History

For high-risk individuals on the basis of factors other than smoking history, the screening effectiveness was obtained from the TALENT study.^17^ The study enrolled 12,011 individuals between 2015 and 2019, with approximately half having a family history of lung cancer. Among the scans performed, 17.4% were positive, leading to a diagnosis of 318 cases of lung cancer in the first round. These were predominantly adenocarcinoma, with a high percentage detected at early stages.^17^ The TALENT study also detected individuals with stage 0 lung cancer, which was typically referred to as “carcinoma-in-situ.” According to expert opinion, stage 0 patients would undergo regular lung cancer postoperation surveillance, which usually consisted of a chest CT every 3 to 6 months in the first 2 years and 6 to 12 months in the next 3 years. The screening was modeled for a single round, as the screening outcomes from the TALENT study were only accessible for the initial round.

### Survival Data

The local lung cancer overall survival data by staging were estimated by the local clinical and epidemiologic experts (Supplementary Table 1). The disease progression-free survival per lung cancer stage at diagnosis was synthesized from various clinical trials, as local retrospective studies reflecting the treatment pathway and patients’ prognosis are not available. For early-stage lung cancer (stage I and II), the primary end point in clinical trials is often set to be disease-free survival, which refers to the survival of patients in complete remission after finishing treatments, as treatments for early-stage lung cancer are with curative intent. Survival data sourced from the ADAURA and IMpower010 clinical trials were used for early-stage lung cancer, encompassing cohorts of patients stratified by the presence or absence of EGFR mutations.^32^^,^^33^ Furthermore, it is noteworthy that both trials exhibited a substantial representation of the Asian population (64% versus 31%), and provided survival data with extensive follow-up time (32.2 mo versus 5 y).^32^^,^^33^ For advanced lung cancer diagnosed at stages III and IV, progression-free survival refers to the survival of patients without tumor growth or cancer spread during or after treatments. Survival data for stage III was obtained from the PACIFIC trial.^34^ For stage IV, results from multiple trials were pooled to synthesize the progression-free survival, using the weights on the basis of the histology and gene mutation prevalence. These trials included KEYNOTE-189,^35^ FLAURA,^36^ and IMpower133,^37^ to cover patients with NSCLC having nonactionable mutations, patients with NSCLC having *EGFR* gene mutations, and patients with SCLC (Supplementary Table 2).

All survival curves were extracted according to the method described by methodology article^39^ and extrapolated in accordance with the NICE DSU Technical Support Document 14.^40^ The curves were first fitted using WebPlotDigitizer to reproduce the corresponding data set. Next, the data were extrapolated in R studio (version 2022.12.0 + 353) (R Core Team, Vienna, Austria) and the parameter values per distribution and the covariance mix were obtained. Moreover, the information criteria will be computed for each distribution to understand which of the standard distributions has the optimal statistical fit for the survival data. The optimal parametric distribution was selected on the basis of the statistical fit, visual inspection, and expert options assessing the clinical plausibility. The parametric distributions selected for each survival curve are delineated in Supplementary Table 3. Background mortality was also considered in the model to account for all-cause mortality, for which HK-specific life tables were used.^41^

### Health Utilities

Utility estimates for patients diagnosed with stage I to IV were on the basis of utility values measured in a previous study utilizing the EQ-5D questionnaire among 1147 patients with lung cancer with performance status scored 0 to 1 on the basis of the Eastern Cooperative Oncology Group score.^42^ In the preprogression state, utility values per stage were 0.85, 0.83, 0.73, and 0.75 respectively. In the postprogression state, utility values were adopted from the corresponding preprogression state. For initial-diagnosed stage I and II patients, a utility value of 0.73 was applied, whereas for initial-diagnosed stage III and IV patients, this value was 0.75, as patients experiencing progressive diseases will have deterioration in their health status and quality of life.^43^ For stage 0 patients with lung cancer and lung cancer-free participants, age-dependent utility values for the general population were generated on the basis of the method delineated in a previous publication.^44^

### Costs

A microcosting approach was taken to capture the resource utilization in the local healthcare settings for LCS. Microcosting is a cost estimation methodology using detailed resource utilization and unit cost data to generate precise estimates of economic costs.^45^ Costs were captured for diagnostic procedures, imaging, surgery, radiotherapy, and systemic therapies, including cytotoxic chemotherapy, targeted therapy, and immunotherapy (Table 2). The unit costs of diagnostic procedures, imaging, hospitalization, and treatments inclusive of the anticancer treatments were obtained from the HK Hospital Authority.^46^ Specifically, the cost estimate for a low-dose CT was determined as the mean of the prevailing costs for low-dose CT thorax quoted by three imaging facilities in HK, and it is estimated at US$254, which is approximately 50% of the cost of a regular contrast-enhanced chest CT (US$507). The corresponding utilization per diagnostic procedure and therapeutic treatment on the basis of the stage of lung cancer was calibrated on the basis of expert opinions (Supplementary Table 1). Resource use and unit costs for lung cancer diagnosis and treatments were subdivided into multiple phases, including diagnosis/postdiagnosis, ongoing, and postprogression phases (Table 2). A detailed description of these phases is provided in Supplementary Table 4. All costs used in the model are expressed in U.S. dollars and are indexed to the year 2023.

**Table 2:** Unit Costs and Health Care Utilization for Lung Cancer Diagnosis and Treatments by Stage at Diagnosis

Part A. Unit Costs and Healthcare Utilization for Lung Cancer Diagnosis and Nonpharmaceutical Therapies for Various Phases													
Intervention	Unit Costs (US$)	Healthcare Utilization^a^											
		Diagnosis/Postdiagnosis^b^				Ongoing				Postprogression^c^			
		Stage I	Stage II	Stage III	Stage IV	Stage I	Stage II	Stage III	Stage IV	Stage I	Stage II	Stage III	Stage IV
Diagnostics and imaging													
Chest x-ray	$24	48.9%	45.2%	53.9%	58.1%	50.9%	47.5%	60.9%	68.5%	75.3%	76.0%	44.2%	57.5%
Contrast-enhanced chest, lower neck, and abdomen CT	$507	40.0%	40.0%	40.0%	30.0%	30.0%	30.0%	30.0%	20.0%	20.0%	20.0%	20.0%	20.0%
PET-CT	$1331	50.0%	50.0%	50.0%	60.0%	5.0%	5.0%	5.0%	15.0%	15.0%	15.0%	15.0%	15.0%
Spirometry	$160	100.0%	100.0%	100.0%	15.0%	0.0%	0.0%	0.0%	10.0%	0.0%	0.0%	0.0%	0.0%
Flexible bronchoscopy alone - no EBUS	$601	40.1%	60.1%	52.1%	42.7%	38.8%	56.0%	36.2%	26.1%	12.8%	8.7%	45.8%	32.9%
EBUS-guided TBNA plus or minus bronchoscopy	$3049	0.1%	0.2%	0.0%	0.1%	0.1%	0.2%	0.0%	0.1%	0.1%	0.0%	0.0%	0.1%
CT biopsy	$970	40.0%	30.0%	40.0%	30.0%	0.0%	0.0%	0.0%	2.0%	0.0%	0.0%	0.0%	0.0%
Surgery													
Lobectomy, wedge resection, pneumonectomy, segmental resection, sleeve resection	$9310	45.1%	28.9%	13.9%	10.1%	41.8%	26.4%	8.7%	7.9%	3.2%	2.9%	19.8%	4.7%
Radiotherapy													
Intracranial radiotherapy	$5414	0.0%	0.0%	0.0%	0.0%	0.0%	0.0%	0.0%	2.0%	0.0%	0.0%	0.0%	20.0%
Radiotherapy for curative intent (SABR)	$6231	0.0%	1.6%	0.2%	0.6%	0.3%	1.5%	1.5%	2.8%	3.8%	5.8%	0.4%	1.9%
Palliative radiotherapy	$1000	0.0%	0.0%	0.0%	5.0%	0.0%	0.0%	0.0%	3.0%	0.0%	0.0%	0.0%	30.0%

Part B. Unit Costs and Healthcare Utilization for Lung Cancer Pharmaceutical Therapies for Postdiagnosis and Postprogress Phases													
Intervention	Unit Costs (US$)^d^	Healthcare Utilization^a^											
		Postdiagnosis^b^					Postprogression^c^						
		Stage I	Stage II	Stage III		Stage IV	Stage I		Stage II	Stage III		Stage IV	
Chemotherapy													
Docetaxel monotherapy	$189	0.0%	0.9%	1.4%		0.4%	8.1%		25.0%	2.3%		0.9%	
Pemetrexed maintenance	$255	0.0%	0.1%	0.5%		0.2%	0.0%		0.0%	0.6%		0.2%	
Chemotherapy doublet													
platinum + vinorelbine (adjuvant in stage II)	$269	0.0%	42.8%	22.5%		5.2%	8.9%		1.8%	21.4%		2.2%	
Gemcitabine + carboplatin	$120	0.0%	22.3%	29.7%		11.1%	33.6%		23.8%	26.9%		10.3%	
Gemcitabine + cisplatin	$159	0.0%	6.3%	7.0%		1.3%	0.9%		2.4%	5.5%		1.1%	
Pemetrexed + platinum	$229	0.0%	26.5%	38.2%		18.6%	47.2%		43.5%	40.8%		20.8%	
Immunotherapy (with or without chemo)													
Pembrolizumab (PD-1)	$6726	0.1%	6.0%	7.2%		2.5%	6.0%		9.5%	8.7%		3.0%	
Durvalumab (PD-1)	$4535	0.0%	1.9%	1.0%		0.1%	0.4%		0.0%	1.3%		0.0%	
Pembrolizumab + carbo/cis + gem/pemetrexed (PD-1)	$5219	0.0%	1.4%	1.4%		0.8%	1.7%		1.8%	1.3%		0.9%	
Targeting therapy with TKI^c^													
Gefitinib (EGFR TKI)	$1084	0.0%	0.0%	0.0%		37.1%	39.8%		27.7%	20.8%		36.0%	
Erlotinib (EGFR TKI)	$83	0.0%	0.0%	0.0%		27.8%	23.2%		26.7%	15.2%		27.9%	
Crizotinib (ALK and ROS1 inhibitor)	$6749	0.0%	0.0%	0.0%		2.4%	1.4%		2.3%	2.5%		2.4%	
Alectinib (ALK TKI)	$6603	0.0%	0.0%	0.0%		1.2%	0.7%		1.6%	1.7%		1.1%	
Osimertinib (EGFR TKI)	$4456	0.0%	0.0%	0.0%		3.0%	4.2%		9.9%	1.0%		3.4%	
Afatinib (EGFR TKI)	$1608	0.0%	0.0%	0.0%		2.7%	2.5%		5.1%	1.3%		2.7%	
Brigatinib (ALK TKI)	$5403	0.0%	0.0%	0.0%		0.0%	0.0%		0.6%	0.3%		0.0%	

Note: The utilization per diagnostic procedure and treatment for lung cancer presented in the table was calibrated on the basis of expert opinions.

CT, computed tomography; EBUS, endobronchial ultrasound; PET-CT, positron emission ultrasound–computed tomography; SABR, stereotactic ablative radiotherapy; TKI, tyrosine kinase inhibitor.

a Percentages listed under “healthcare utilization” represent the proportion of patients within each lung cancer stage who received the specified diagnostic or treatment intervention during the corresponding phase of care.

b Postdiagnosis refers to the initial postdiagnosis treatments received after a diagnosis, usually covering the first 6 months.

c Postprogression’ refers to the phase of recurrence or progression of the disease, signified by the need for the second-line treatments. Resources utilization for this phase were collected for the initial 6 months after recurrence or progression of the disease.

d Unit costs per pharmaceutical therapy are defined as standard dosing used as per drug label/clinical practice guidelines over a 30-day period.

### Sensitivity Analyses

One-way sensitivity analysis was conducted with deterministic changes of plus or minus 20% of the base-case parameter values. Results were used to identify the main drivers for the cost-effectiveness of LCS for both cohorts—that is, high-risk individuals on the basis of smoking history and high-risk individuals identified by criteria beyond smoking history. In addition, tornado diagrams were plotted to illustrate the variations in ICERs within each cohort and enhance the interpretability of parameter-specific impacts. A probabilistic sensitivity analysis was conducted by simulating the whole cohort with a set of parameter values sampled probabilistically from distributions reflecting parameter uncertainties. Utility values adhered to the beta distribution, cost parameters followed the gamma distribution, and the distributions applied to other parameters are elucidated in Table 1.

### Scenario Analyses

Scenario analyses were undertaken to investigate the cost-effectiveness of LCS under various conditions, such as varying LCS uptake rates for the target population, inclusion of a screening-associated disutility,^18^ analyses with different time horizons and discounting rates, and analyses with different LCS rounds.

## Results

### Base-Case Results

Over a lifetime horizon, the ICER for high-risk individuals on the basis of smoking history was US$14,122 per QALY, with incremental costs and QALYs of US$331 million and 23,436, respectively. The ICER for high-risk individuals on the basis of factors other than smoking history was US$9610 per QALY, with incremental costs and QALYs of US$ 97 million and 10,063, respectively (Table 3). In addition, the ICER per LYG for these two high-risk cohorts was US$10,486 and US$7272, respectively. Moreover, compared with no screening, screening led to 5162 additional stage I lung cancer detected and 1580 lung cancer deaths averted, among high-risk individuals on the basis of smoking history. Meanwhile, for high-risk individuals on the basis of factors other than smoking history, these figures were 1625 and 482, respectively (Table 3).

**Table 3:** Base-Case Results of Lung Cancer Screening for High-Risk Individuals

Base-case Results for High-Risk Individuals on the Basis of Smoking History			
	Screening	No Screening	Incremental (screened vs nonscreened)
**Clinical and health outcomes**			
**Lung cancer diagnoses**			
Total	19,697 (100%)	18,680 (100%)	1017
Stage I	8396 (43%)	3235 (17%)	5162
Stage II	1154 (6%)	890 (5%)	264
Stage III	2865 (15%)	2729 (15%)	136
Stage IV	7283 (37%)	11,827 (63%)	–4544
Stage IV averted	4544		
**Lung cancer deaths**			
Total	13,750	15,330	–1580
Stage I	4320	1664	2656
Stage II	712	549	163
Stage III	1542	1464	78
Stage IV	7176	11,653	–4477
**Life years gained (LYGs)**			
Total	2,036,301	2,004,741	31,560
Stage I	63,353	24,365	38,988
Stage II	7101	5423	1678
Stage III	28,991	27,428	1563
Stage IV	8454	13,721	–5267
LC free participants	1,928,403	1,933,804	–5401
**Quality-adjusted life years (QALYs)**			
Total	1,654,571	1,631,135	23,436
Stage I	47,671	18,333	29,339
Stage II	5390	4115	1276
Stage III	21,250	20,102	1148
Stage IV	6333	10,278	–3945
LC free participants	1,573,927	1,578,308	–4381
**Costs (US$)**			
Total	$637,191,905	$306,236,801	$330,955,103
Screening costs	$248,411,526	N.A.	$248,411,526
Diagnostic costs	$99,591,903	$90,252,611	$9,339,292
Treatment costs	$289,188,475	$215,984,190	$73,204,285
Stage I	$137,643,249	$52,943,619	$84,699,630
Stage II	$20,815,767	$15,906,221	$4,909,546
Stage III	$96,116,327	$90,956,775	$5,159,552
Stage IV	$34,613,132	$56,177,574	$–21,564,442
**Health economic outcomes**			
ICER per LYG	$10,486	NMB^a^	N.A.
ICER per QALY	$14,122	NMB^a^	$238,587,630 – $611,220,725

**Base-case results for high-risk individuals identified by criteria beyond smoking history**			

	**Screening**	**No screening**	**Incremental (screened vs nonscreened)**

**Clinical and health outcomes**			
**Lung cancer diagnoses**			
Total	2853 (100%)	1615 (100%)	1238
Stage 0^b^	427 (N.A.)	N.A.	N.A.
Stage I	1904 (67%)	280 (17%)	1625
Stage II	71 (2%)	77 (5%)	-6
Stage III	175 (6%)	236 (15%)	-61
Stage IV	702 (25%)	1022 (63%)	-320
Stage IV averted	320		
**Lung cancer deaths**			
Total	1827	1345	482
Stage I	984	144	839
Stage II	44	48	–4
Stage III	105	142	–37
Stage IV	695	1011	–317
**Life years gained**			
Total	214,632	201,333	13,299
Stage I	17,973	2639	15,334
Stage II	543	586	–44
Stage III	2155	2906	–751
Stage IV	953	1387	–434
LC free participants^c^	193,008	193,814	–806
**Quality-adjusted life years**			
Total	177,304	167,241	10,063
Stage I	13,651	2,005	11,646
Stage II	416	450	–33
Stage III	1585	2138	–552
Stage IV	714	1040	–326
LC free participants^c^	160,938	161,610	–672
**Costs**			
Total	$128,330,627	$31,622,448	$96,708,179
Screening costs	$23,159,089	N.A.	$23,159,089
Diagnostic costs	$54,148,277	$9,029,013	$45,119,263
Treatment costs	$51,023,261	$22,593,434	$28,429,827
Stage I	$38,453,537	$5,646,932	$32,806,605
Stage II	$1,573,755	$1,700,193	$-126,438
Stage III	$7,102,353	$9,577,269	$–2,474,916
Stage IV	$3,893,615	$5,669,040	$–1,775,425
Health economic outcomes			
ICER per LYG	$7272	NMB^a^	N.A.
ICER per QALY	$9610	NMB^a^	$147,846,132 – $308,252,507

ICER, incremental cost-effectiveness ratio; LC, lung cancer, LYG, life years gained; N.A., not applicable; NMB, net monetary benefit; QALY, quality-adjusted life years.

a NMB refers to net monetary benefit, calculated by multiplying the health gain (e.g., QALYs) by the willingness-to-pay threshold and subtracting the cost of the intervention, indicating whether a healthcare intervention provides good value for money compared with alternatives.

b Stage 0 was only detected in the screening arm; to ensure comparability of lung cancer stage distribution between the screening and no screening arms, stage 0 is not presented as a percentage. N.A. (not applicable) is shown instead.

c Lung cancer–free participants refer to the lung cancer screening participants who do not have lung cancer.

### Sensitivity Analyses

**Figure 1:**
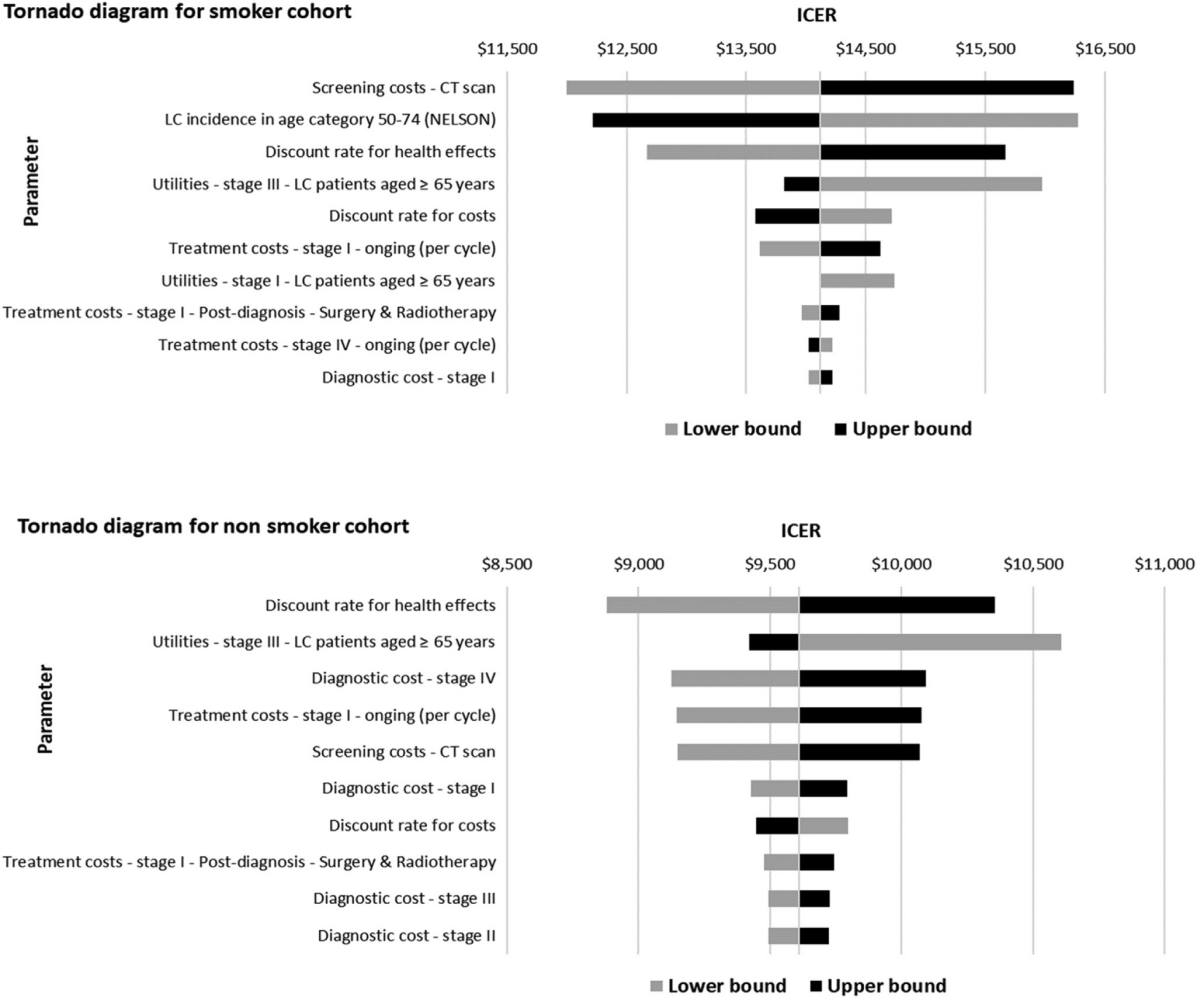
Tornado diagrams from the one-way sensitivity analysis. The diagrams illustrate the impact of varying key input parameters on the ICER of lung cancer screening compared *with* no screening. Parameters are ranked by the magnitude of their influence on the ICER, with the most influential at the top. Bars extending to the left or right indicate the direction and extent of change in the ICER when the parameter is varied across its plausible range. Smoking cohort refers to high-risk individuals on the basis of smoking history. Non smoker cohort refers to high-risk individuals on the basis of factors other than smoking history. ICER, incremental cost-effectiveness ratio; LC, lung cancer; CT, computed tomography.

**Figure 2:**
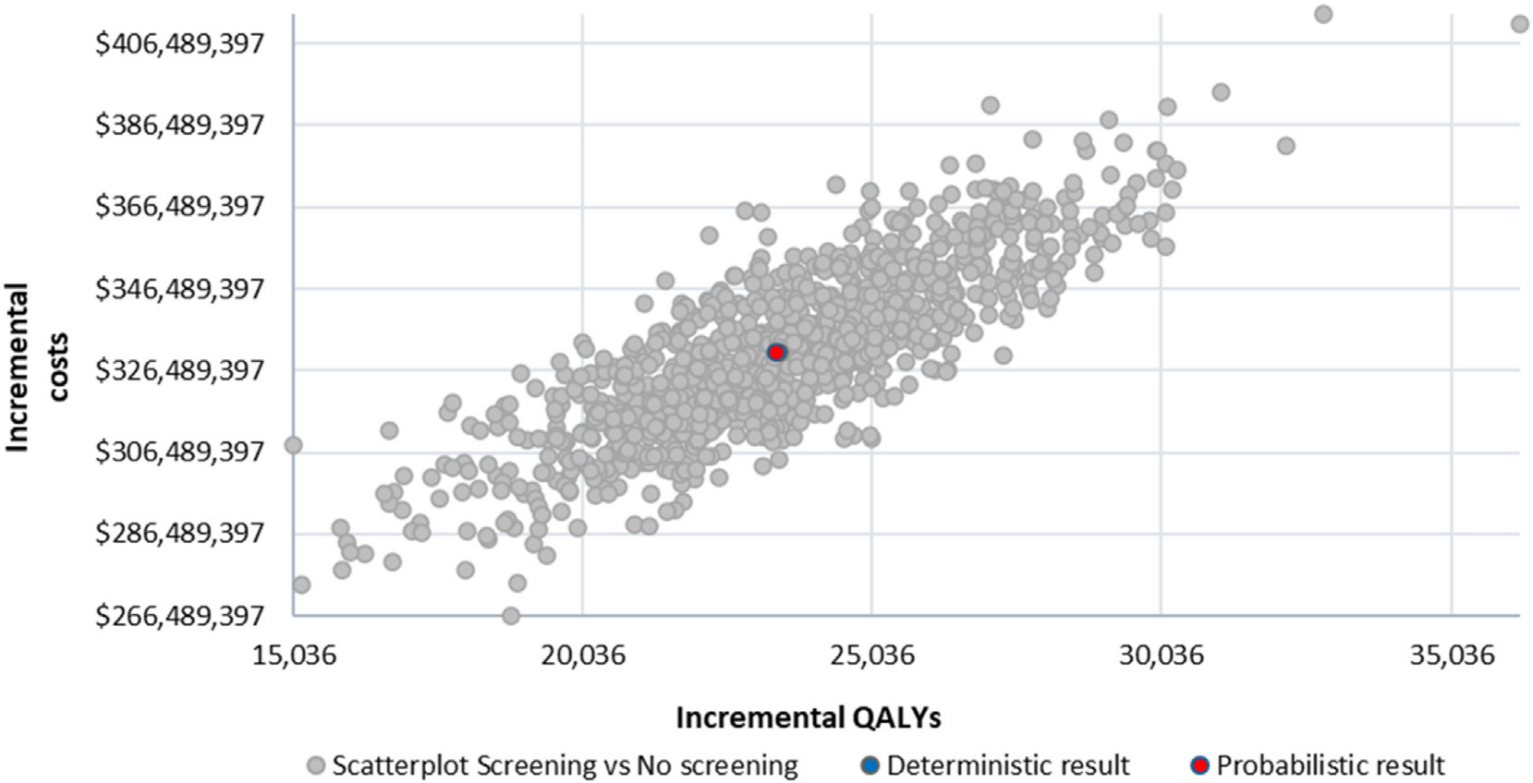
The incremental cost-effectiveness scatterplot for high-risk individuals on the basis of smoking history. Each point represents a simulation from the probabilistic sensitivity analysis, illustrating the difference in costs and QALYs between the screened and nonscreened groups. The scatterplot illustrates the uncertainty around the estimated incremental cost-effectiveness ratio for the screening strategy. QALY, quality-adjusted life years.

**Figure 3:**
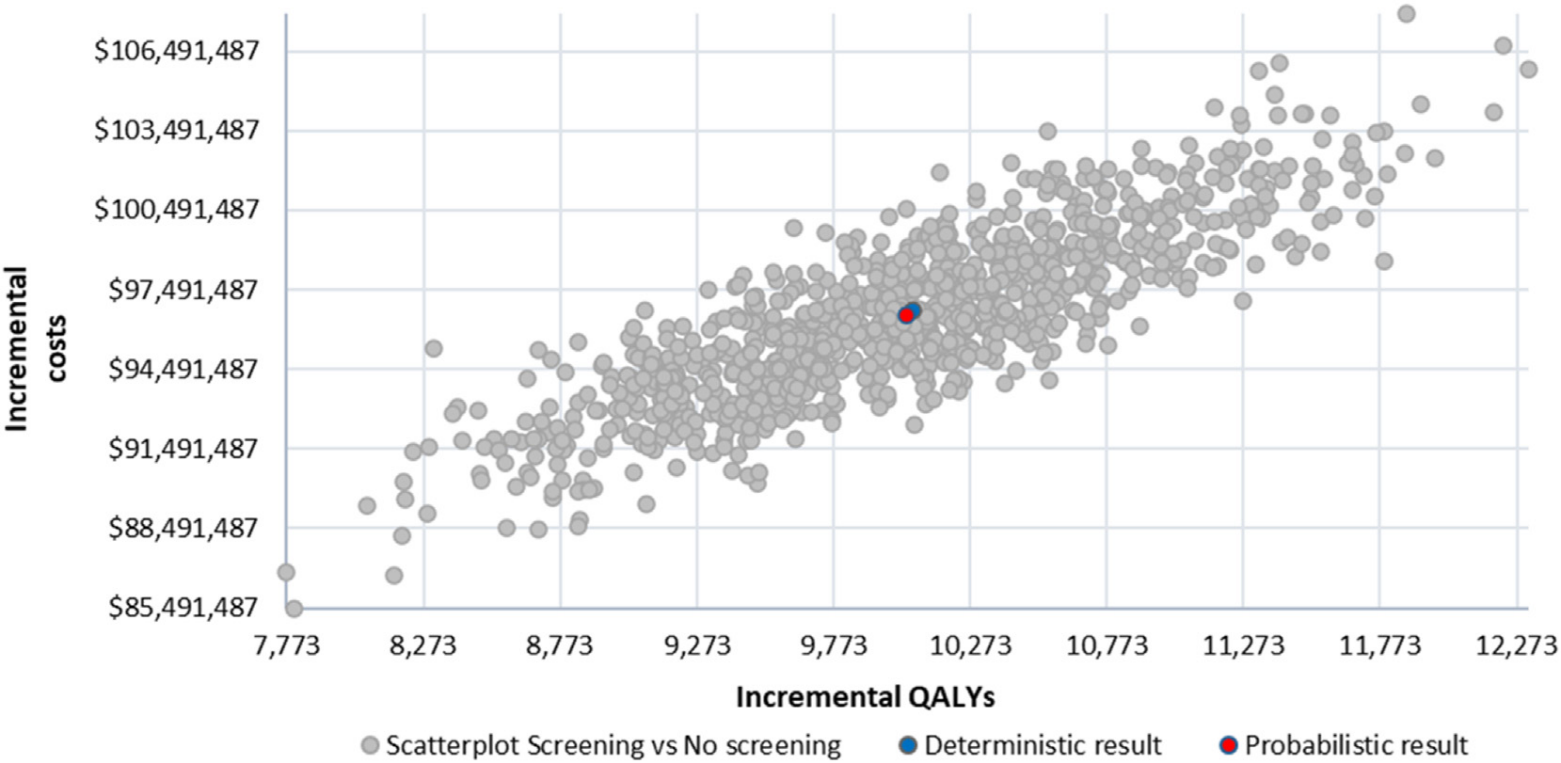
The incremental cost-effectiveness scatterplot for high-risk individuals on the basis of factors other than smoking history. Each point represents a simulation from the probabilistic sensitivity analysis, showing the difference in costs and QALYs between the screened and nonscreened groups. The scatterplot illustrates the uncertainty around the estimated incremental cost-effectiveness ratio for the screening strategy.

One-way sensitivity analysis resulted in small changes in the ICER, all within HK WTP thresholds (Fig. 1). For high-risk individuals on the basis of smoking history, the main variation influencing the ICER came from changes in LDCT unit costs, lung cancer incidence in the population aged 50 to 74 years, and discount rates for the health effect. For high-risk individuals on the basis of factors other than smoking history, the main variation influencing the ICER stemmed from changes in discount rates for the health effect, utility values for stage III patients, and diagnostic costs for stage IV patients. On the basis of 1000 iterations, the probabilistic sensitivity analysis resulted in an average ICER of US$14,152 per QALY for high-risk individuals on the basis of smoking history and an average ICER of US$9616 per QALY for high-risk individuals on the basis of factors other than smoking history. Figures 2 and 3 illustrate the spread of probabilistic simulations in the form of an incremental cost-effectiveness scatterplot. All simulations resulted in an ICER under the WTP threshold for HK.

### Scenario Analyses

Results for scenario analyses are presented in Table 4. Increasing the screening uptake rate would result in more QALYs gained per patient with lung cancer. In addition, reductions in LDCT costs, diagnostic costs, and treatment costs resulted in lower ICERs compared with the base-case estimates. All scenarios resulted in an ICER well below HK’s WTP threshold of US$24,302 to US$40,202 per QALY, except for the results under a 5-year and 10-year time horizon for high-risk individuals on the basis of smoking history (US$88,814 and US$51,523 per QALY, respectively).

**Table 4:** Scenario Analyses Results for Lung Cancer Screening Under Various Situations

Scenario	Smoking cohort^a^		Nonsmoking cohort^b^	
	ICER	QALYs gained per patient	ICER	QALYs gained per patient
Base-case results	$14,122	1.27	$9,610	2.25
Screening uptake rate (25%)	$14,122	0.70	$9,610	1.51
Screening uptake rate (75%)	$14,122	1.98	$9,610	2.87
Time horizon (5 y)	$88,814	0.28	$27,129	0.61
Time horizon (10 y)	$51,523	0.44	$16,773	1.04
Time horizon (15 y)	$25,897	0.71	$13,147	1.42
Discount rates (cost: 0%, health outcome: 0%)	$9952	2.17	$6984	3.62
Discount rates (cost: 6%, health outcome: 6%)	$17,636	0.92	$11,695	1.72
Include screening-associated disutility	$15,204	1.27	$9856	2.25
Increase background mortality by 100%	$16,429	1.08	$10,652	1.94
Varying weights used for stage I&II D/PFS (EGFR 10 %, nonEGFR 90%)	$13,827	1.27	$9372	2.27
Varying weights used for stage I&II D/PFS (EGFR 25 %, nonEGFR 75%)	$13,974	1.27	$9491	2.26
Use the overall survival data published by IASLC^c^	$9978	1.96	$7187	4.71
Cut down unit costs for low-dose CT scans - US$191	$11,502	1.27	$9041	2.25
Cut down unit costs for low-dose CT scans - US$127	$8842	1.27	$8464	2.25
Cut down unit costs for low-dose CT scans - US$63	$6,182	1.27	$7886	2.25
Half diagnostic costs for stage I - US$836	$13,874	1.27	$9155	2.25
Half diagnostic costs for stage IV - US$3921	$14,365	1.27	$8402	2.25
Half treatment costs for the ongoing phase for stage I patients - US$243	$12,862	1.27	$8446	2.25
Half treatment costs for the ongoing phase for stage IV patients - US$411	$14,369	1.27	$9658	2.25

CT, computed tomography; D/PFS, disease/progression-free survival; IASLC, International Association for the Study of Lung Cancer; ICER indicates incremental cost-effectiveness ratio; QALY, quality-adjusted life years.

a Smoking cohort refers to high-risk individuals on the basis of smoking history.

b Nonsmoking cohort refers to high-risk individuals on the basis of factors other than smoking history.

c The overall survival data used in this scenario is published by IASLC (Goldstraw et al, 2016).

## Discussion

Our study found that LCS with LDCT targeting individuals with a high risk of developing lung cancer, on the basis of smoking history and factors other than smoking history, would lead to more LYG and a mortality reduction in HK. In addition, the ICERs per QALY are well below the WTP threshold for both cohorts. This study presents the initial endeavor to evaluate the cost-effectiveness of LDCT screening in HK. It also stands as one of the few pioneering studies, investigating clinical and health economic outcomes of incorporating high-risk individuals on the basis of factors other than smoking history into LCS, aiming to adequately reflect the epidemiologic profile for patients with lung cancer in HK.^13^

The cost-effectiveness of LCS for high-risk individuals on the basis of smoking history has been analyzed in various studies. Integration of the most recent findings from the NELSON study has revealed that LDCT screening is cost-effective across various nations.^21^^,^^47^ For example, the U.K. National Screening Committee reported that annual LDCT screening, targeting heavy smokers aged 55 to 75 years with a risk greater than 1.5%, exhibited an ICER of £8517 per QALY, which at the same time yielded significant clinical benefits.^48^ On the basis of these results, the U.K. National Health Service recommended targeted screening for lung cancer through the Targeted Lung Health Check Programme, which focuses on heavy smokers. Our study estimated that screening high-risk individuals on the basis of smoking history in HK resulted in an ICER of US$14,122 per QALY, which is higher than the figure reported by the U.K. National Screening Committee. This might be mainly explained by the relatively higher costs of an LDCT scan (US$254 in HK versus US$93 in the UK), given that the LDCT cost was ranked as the most influential parameter for the ICER according to the sensitivity analysis in our study. Furthermore, scenario analysis revealed that the ICER would decrease with a reduction in CT scan costs (Table 4).

A previous study conducted in the People’s Republic of China concluded that LDCT screening in the general population, including never-smokers, could be cost-effective for men with a 75% probability, but not for women.^49^ However, these studies used screening findings and inclusion criteria from the NLST. Applying only the NLST criteria would miss more than 80% of high-risk LCS participants in the People’s Republic of China.^50^ To address this issue, our model used the inclusion criteria and screening outcomes from the TALENT study to account for the risk factors beyond smoking history, such as lung cancer family history, exposure to passive smoking, tuberculosis or chronic pulmonary disease history, and high cooking index.^17^ Consequently, the ICER of implementing LCS for high-risk individuals identified by criteria other than smoking history was US$9610 per QALY. This provides a reference for the cost-effectiveness of LCS in other East Asian populations deemed high risk of developing lung cancer on the basis of factors other than smoking history. Further studies may warrant a verification of our findings using data from other East Asian countries with similar LC epidemiologic and demographical profiles.

Population-based cancer screening has been recommended for cervical cancer, colorectal cancer, and breast cancer in HK, with different interventions and varying ICERs.^51^^,^^52^ Specifically, colorectal cancer screening in eligible populations with colonoscopy every 10 years, or with sigmoidoscopy every 5 years, has been recommended with ICERs being US$55,369 and US$108,879 per QALY, respectively.^51^

Unlike the United Kingdom and the United States where there are established benchmarks for WTP thresholds,^24^ there is no official WTP threshold for health services in HK. We have adopted a WTP threshold of US$24,302 to US$40,202 per QALY for this study, and it was informed by a study reporting WTP thresholds on the basis of estimates of opportunity costs, which reflected the health opportunity costs within different health care systems, offering a realistic benchmark for determining the value of money, particularly in the absence of an officially defined local threshold.^25^ Our ICER estimates are below the WTP set in the study and also below the ICER thresholds used from the previous decisions on recommending cancer screening programs in HK.^51^

Scenario analyses revealed that a decrease in the expenditures associated with treating patients with early-stage lung cancer contributed to a reduction in the ICER for LDCT screening. In our study, the microcosting method was used to capture the resources used. Unlike traditional costing methods that rely on aggregated data or average costs, microcosting involves a thorough examination of all relevant resources and their associated costs to provide a more accurate and precise estimation.^45^ Notably, the costs for novel treatments, such as immunotherapy and targeted therapy, were taken into account to reflect the up-to-date treatment regimens. Nevertheless, the model did not encompass the initial setup costs and ongoing operational expenses for the population-level LCS program. However, this limitation may have a minimal impact on the overall cost-effectiveness assessment, as HK has a well-established health care infrastructure. Given the existing resources and health care capacity, substantial investments to initiate the LCS are unlikely to be required, making these costs relatively negligible. Another limitation was the lack of data for stage 0 patients detected in the nonsmoker cohort on the basis of the TALENT study.^17^ These patients were assumed to follow the general population’s survival and quality of life after the surgery, as a conservative estimate. Further research is needed to provide more evidence on the overall survival, utility values, and relevant costs for this so-called “carcinoma-in-situ” cohort.

The assessment of the costs, benefits, and cost-effectiveness of LCS in individuals without a history of smoking remains one of the prevailing controversies. Admittedly, there are ongoing debates regarding this population group, primarily because of concerns of potential overdiagnosis, false-positive detections, and radiation exposure. In addition, the potential bias in survival statistics—such as lead-time bias, in which early detection may artificially inflate survival rates without a corresponding reduction in mortality—has also been widely discussed. Accordingly, there is a need to investigate this topic through prospective clinical trials or rigorous analyses of real-world evidence to address these concerns.

In conclusion, LCS with LDCT can be considered cost-effective in HK for high-risk individuals on the basis of smoking history and factors other than smoking history, contributing to the health benefits of the population. Our findings support a population-based LCS for all high-risk individuals identified through criteria beyond smoking history. These findings may also be applicable to other East Asian countries with similar lung cancer epidemiologic and demographical profiles.

## CRediT Authorship Contribution Statement

**Herbert Ho-fung Loong**: Conceptualization, Methodology, Data curation, Validation, Writing - review and editing.

**Xuanqi Pan**: Conceptualization, Methodology, Data curation, Software, Visualization, Writing – original draft.

**Carlos K. H. Wong**: Conceptualization, Methodology, Data curation, Writing - review and editing.

**Chao-Hua Chiu**: Data curation, Validation.

**Szu-Chun Yang**: Methodology, Validation, Writing - review and editing.

**Matthew Shing Hin Chung**: Methodology, Data curation.

**Molly Siu Ching Li**: Validation, Writing - review and editing.

**Lisa de Jong**: Methodology, Validation, Supervision, Writing - review and editing.

**Harry Groen**: Validation, Supervision, Writing - review and editing.

**Maarten J. Postma**: Methodology, Validation, Supervision, Writing - review and editing.

**Pan-Chyr Yang**: Conceptualization, Methodology, Data curation, Validation, Supervision

## Disclosure

Dr. Wong reports receiving funding from AstraZeneca Hong Kong Limited, and Dr. SC Yang reports receiving grants from the 10.13039/100020595National Science and Technology Council, Taiwan (110-2314-B-006-100-MY2 and 112-2314-B-006-013-MY2). The remaining authors declare no conflict of interest.
