# Supplementary material for: Incorporating High-Risk Individuals Beyond Smoking History Into Lung Cancer Screening in Hong Kong: A Cost-Effectiveness Study
Source: JTO Clin Res Rep. 2025 Jun 13;6(10):100860. doi: 10.1016/j.jtocrr.2025.100860 (PMC12570320; doi:10.1016/j.jtocrr.2025.100860)
Supplement: Supplementary Figure 1 and Supplementary Table 1-4 [file mmc1.pdf]

Supplementary Materials

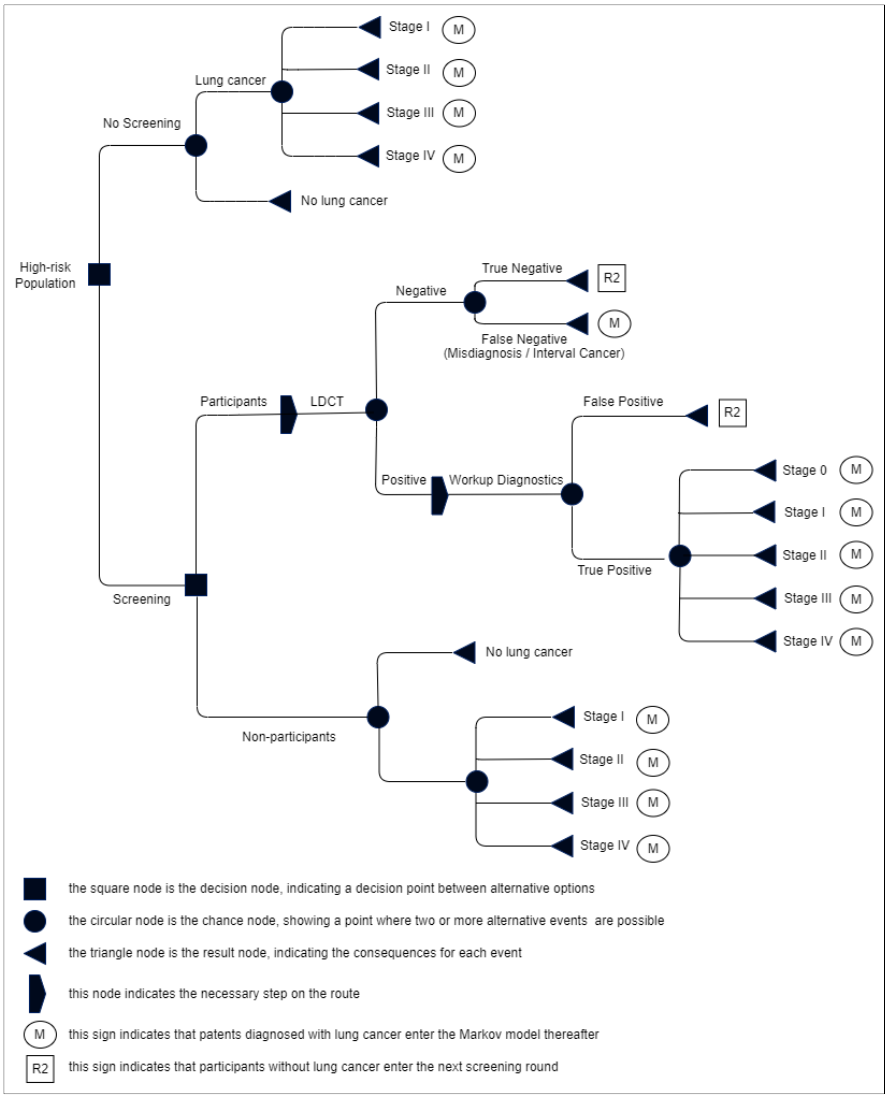

Figure S1. Decision tree for lung cancer screening based on the TALENT study for non-smokers.

Table S 1. Details for the experts panel.

Name	Affiliate
Prof. Herbert Ho-fung Loong	Department of Clinical Oncology, Faculty of Medicine, The Chinese University of Hong Kong, Hong Kong SAR, China
Prof. Carlos K H Wong	Department of Pharmacology and Pharmacy, LKS Faculty of Medicine, University of Hong Kong, Hong Kong SAR, China
Matthew Shing Hin Chung	Department of Pharmacology and Pharmacy, LKS Faculty of Medicine, University of Hong Kong, Hong Kong SAR, China

Table S 2. Clinical trials and corresponding weights used to synthesize the progression-free survival data for stage IV lung cancer patients.

Trial	Patients enrolled	Intervention	Trial design	Weight [a]
KEYNOTE-189 (35)	616 non-actionable mutations NSCLC patients (advanced stage)	Pembrolizumab plus chemotherapy	Phase III double blind RCT	68%
FLAURA (36)	556 patients with previously untreated, EGFR mutation-positive advanced NSCLC	Osimertinib	Phase III double blind RCT	17%
IMpower133 (37)	403 SCLC patients (advanced stage)	Atezolizumab plus chemotherapy	Phase III double blind RCT	15%

NSCLC, non-small cell lung cancer; RCT, randomized control trial; EGFR, epidermal growth factor receptor; SCLC, small cell lung cancer.

[a] the weights used to synthesize the survival curves from various clinical trials were based on the epidemiology for lung cancer patients and the prevalence of the gene mutation.

Table S 3. Parametric distributions and the corresponding parameters used to extrapolate survival curves.

**Parametric distribution per sub-stage**	**Base-case value**	**Reference**
**Overall survival**		
Stage I (generalized gamma distributed - mu)	3.6707	Experts opinions *
Stage I (generalized gamma distributed - sigma)	1.7467	Experts opinions
Stage I (generalized gamma distributed - Q)	-0.5483	Experts opinions
Stage II (Weibull distributed - gamma)	0.6071	Experts opinions
Stage II (Weibull distributed - lambda)	62.5513	Experts opinions
Stage III (generalized gamma distributed - mu)	1.4025	Experts opinions
Stage III (generalized gamma distributed - sigma)	0.9577	Experts opinions
Stage III (generalized gamma distributed - Q)	-1.7354	Experts opinions

Stage IV (Log-logistic distributed - kappa)	1.3928	Experts opinions
Stage IV (Log-logistic distributed - theta)	7.0448	Experts opinions
**Disease/progression-free survival**		
Stage I-II EGFR mutated (Log-normal distributed - mu)	2.9510	(32)
Stage I-II EGFR mutated (Log-normal distributed - delta)	1.1445	(32)
Stage I-II nonEGFR mutated (Log-normal distributed - mu)	3.55331	(33)
Stage I-II nonEGFR mutated (Log-normal distributed - delta)	1.50003	(33)
Stage III - Durvalumab arm (Weibull distributed - gamma) [+]	0.9134	(34)
Stage III - Durvalumab arm (Weibull distributed - lambda)	22.7957	(34)
Stage III - Control arm (Log-normal distributed - mu) [+]	1.9114	(34)
Stage III - Control arm (Log-normal distributed - delta)	1.2816	(34)
Stage IV - non-EGFR mutated NSCLC (Log-logistic distributed - kappa)	1.5881	(35)
Stage IV - non-EGFR mutated NSCLC (Log-logistic distributed - theta)	8.5885	(35)
Stage IV - EGFR mutated NSCLC (Log-normal distributed - mu)	2.8941	(36)
Stage IV - EGFR mutated NSCLC (Log-normal distributed - delta)	0.9089	(36)
Stage IV - SCLC (Log-logistic distributed - kappa)	2.5367	(37)
Stage IV - SCLC (Log-logistic distributed - theta)	5.2757	(37)

EGFR, epidermal growth factor receptor; NSCLC, non-small cell lung cancer; SCLC, small cell lung cancer.

[*] *The local lung cancer overall survival data by staging were estimated by the local clinical and epidemiological experts.*

[+] *for lung cancer stage III, survival data from both the intervention arm (treated with* Durvalumab*) and the control arm was used, and it was obtained from the trial PACIFIC; these survival data was weighted based on the number of patients enrolled in both arms.*

Table S 4. Description of different phases used to synthesize diagnostic and treatment costs for lung cancer patients.

Phase	Description
Diagnosis/post-diagnosis phase	It referred to the diagnostic procedures received to establish a diagnosis for the diagnostics and imaging session in Table 2; while for surgery and radiotherapy sessions in Table 2, it referred to the initial post-diagnosis treatments received after a diagnosis, which usually covered the first six months.
Ongoing phase	It referred to the regular check-ups and monitoring received after the initial treatments, and it was collected per three months. These costs were allocated over a five-year period following the initial diagnosis for early-stage lung cancer patients. This duration was chosen based on the assumption that patients would benefit from early detection and subsequently achieved clinical recovery. Conversely, for patients with advanced-stage lung cancer, the costs were accumulated until the point of their eventual demise. This

	approach acknowledges the differing disease trajectories and prognoses between early-stage and advanced-stage patients, thereby appropriately reflecting the resource utilization and associated costs over the respective timeframe.
Post-progression phase	It referred to the stage characterized by disease recurrence or progression of the disease, signified the need for the second-line treatments among patients. During this phase, resource consumption data was gathered for the initial six-month period following the occurrence of disease recurrence or progression.
