# Supplementary material for: Incorporating High-Risk Individuals Beyond Smoking History Into Lung Cancer Screening in Hong Kong: A Cost-Effectiveness Study
Source: JTO Clin Res Rep. 2025 Jun 13;6(10):100860. doi: 10.1016/j.jtocrr.2025.100860 (PMC12570320; doi:10.1016/j.jtocrr.2025.100860)
Supplement: Supplementary Material [file mmc2.docx]

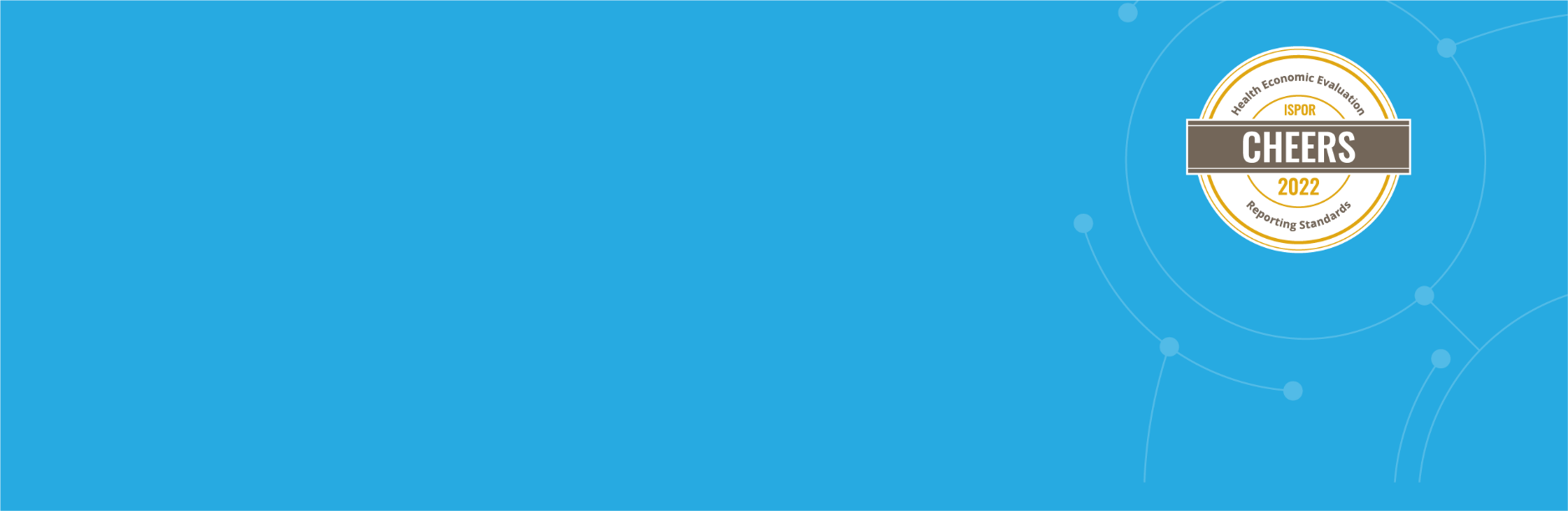


**Consolidated Health Economic Evaluation Reporting Standards
(CHEERS) 2022 Checklist**

The CHEERS 2022 statement replaces the 2013 CHEERS statement, which should no longer
be used. The CHEERS 2022 checklist contains 28 items with accompanying descriptions.
Checklist users should indicate the section of the manuscript where relevant information
can be found. The authors recommend using a section heading with a paragraph number.
If an item does not apply to a particular economic evaluation, checklist users are encouraged
to report “Not Applicable.” If information is otherwise not reported, checklist users are encouraged to

write, “Not Reported.” Users should avoid the term “Not Conducted” as CHEERS is intended to guide and

capture reporting. Additional information on CHEERS 2022 can be found here.

**Title**

**1. Title**

Identify the study as an economic evaluation and specify the interventions being compared.

Yes, the title is "Incorporating high-risk individuals beyond smoking history into lung cancer screening in Hong Kong: A cost-effectiveness study", which identifies the study as an economic evluation (a cost-effectiveness study), also specify the intervention being lung cancer screening.

**Abstract**

**2. Abstract**

Provide a structured summary that highlights context, key methods, results, and alternative analyses.

Yes, the structured abstract has highlighted the centext, key methods, results, and also alternative analysese for the study.

**Introduction**

**3. Introduction: Background and Objectives**

Give the context for the study, the study question, and its practical relevance for decision making in policy or practice.

Yes, the introduction gives the background for the study, presenting the context for the study, as well as the study question, also demonstrate the pratical relevance for the decision making in policy and practice. In the study, it states though the cost-effectiveness of lung cancer screening (LCS) with low-dose CT targeting populations with significant history of smoking has been investigated, the cost-effectiveness of LCS for a mixed cohort, consisting of high-risk individuals based on smoking history and on factors other than smoking history, has yet to be explored. Therefore, this modelling study aimed to investigate whether LCS is cost-effective when incorporating such a mixed screening cohort

**Methods**

**4. Health economic analysis plan**

Indicate whether a health economic analysis plan was developed and where available.

Yes, in the Method session, the model structure and model inputs for the cost-effectiveness analysis were described comprehensively.

**5. Study population**

Describe characteristics of the study population (such as age range, demographics, socioeconomic, or clinical characteristics).

Yes, in the Method session, in the "Eligible population" sub-session, the study population was described, and the relevant data was also presented in Table 1.

**6. Setting and location**

Provide relevant contextual information that may influence findings.

Yes, authors provided the relevant information for the setting and location, the study was conducted in the HongKong setting, and took a healthcare provider perspective.

**7. Comparators**

Describe the interventions or strategies being compared and why chosen.

Yes, the study describes the intervention being investigated as the LCS with low-dose CT, and it is compared to no screening, as the current standard of care in HongKong.

**8. Perspective**

State the perspective(s) adopted by the study and why chosen.

Yes, the study states that it takes a healthcare provider perspective, as LCS is a public healthcare program, which is to be funded by the government.

**9. Time horizon**

State the time horizon for the study and why appropriate.

Yes, the study states that it takes a lifetime horizon to fully capture the long-term health benefits and costs.

**10. Discount rate**

Report the discount rate(s) and reason chosen.

Yes, the study states that a 3.5% annual discount rate was used for both health outcomes and monetary outcomes, accoring to the guideline.

**11. Selection of outcomes**

Describe what outcomes were used as the measure(s) of benefit(s) and harm(s).

Yes, the study states that "The primary health outcomes were the quality-adjusted life years (QALYs) and life years gained (LYG). "

**12. Measurement of outcomes**

Describe how outcomes used to capture benefit(s) and harm(s) were measured.

Yes, in the Method session, the sub-session "Survival data" and "Health utilities" describe how the outcomes were measured.

**13. Valuation of outcomes**

Describe the population and methods used to measure and value outcomes.

Yes, in the Method session, the population and methods used to measure and value outcomes were described.

**14. Measurement and valuation of resources and costs**

Describe how costs were valued.

Yes, in the Method session, the sub-session "Costs" describes how the costs were valued.

**15. Currency, price date, and conversion**

Report the dates of the estimated resource quantities and unit costs, plus the currency and year of conversion.

Yes, the study states in the Method session that "All costs used in the model are expressed in US dollars and are indexed to the year 2023."

**16. Rationale and description of model**

If modeling is used, describe in detail and why used. Report if the model is publicly available and where it can be accessed.

Yes, in the Method session, the sub-session "model structure" describes that a decision tree and a state-transition Markov model are used for the modelling, details can be found in a prior publicaiton X Pan et al 2024.

**17. Analytics and assumptions**

Describe any methods for analyzing or statistically transforming data, any extrapolation methods, and approaches for validating any model used.

Yes, in the Method session, the sub-session "survival data" described the extrapolation method used to extrapolate the survival data for lung cancer patients to the lifetime horizon, and it is in accordance with the NICE DSU Technical Support Document 14.

**18. Characterizing heterogeneity**

Describe any methods used for estimating how the results of the study vary for subgroups.

Yes, the aim of the study is to investigate the cost-effectiveness for LCS with low-dose CT for a mixed cohort, including both the high-risk individuals based on smoking history and high-risk individuals based on criteria beyond smoking history. Therefore, the study naturally estimate the results for subgroups.

**19. Characterizing distributional effects**

Describe how impacts are distributed across different individuals or adjustments made to reflect priority populations.

Yes, a probabilistic sensitivity analysis (PSA) was conducted to account for the distributional effects, and address the parameter uncertainties.

**20. Characterizing uncertainty**

Describe methods to characterize any sources of uncertainty in the analysis.

Yes, the study has conducted both one-way sensitivity analysis (OSA) and probabilistic sensitivity analysis (PSA) to address the uncertainties, and results are presented in Figure 1, 2, and 3.

**21. Approach to engagement with patients and others affected by the study**

Describe any approaches to engage patients or service recipients, the general public, communities, or stakeholders (eg, clinicians or payers) in the design of the study.

Yes, in Method session, it states that the scenario analyses have been conduct to investigate the impact of varying LCS uptake rate on the results, and in Results session, it reports that "Increasing the screening uptake rate would result in more QALYs gained per lung cancer patient".

**Results**

**22. Study parameters**

Report all analytic inputs (eg, values, ranges, references) including uncertainty or distributional assumptions.

Yes, the study has reported all analytic inputs in the Methods session with both text and tables (Table 1 and 2).

**23. Summary of main results**

Report the mean values for the main categories of costs and outcomes of interest and summarize them in the most appropriate overall measure.

Yes, the study has reported both the clinical outcomes and health economic outcomes in the Results session both with text and the table (Table 3). In addition, the mean values for the main categories of costs and outcomes of interest were summarized in the most appropriate overall measure.

**24. Effect of uncertainty**

Describe how uncertainty about analytic judgments, inputs, or projections affects findings. Report the effect of choice of discount rate and time horizon, if applicable.

Yes, the study has conducted both one-way sensitivity analysis (OSA) and probabilistic sensitivity analysis (PSA) to address the uncertainties (Figure 1, 2, and 3). In addition, scenario analyses were conducted to report on the effect of choice of discount rate and time horizon (Table 4).

**25. Effect of engagement with patients and others affected by the study**

Report on any difference patient/service recipient, general public, community, or stakeholder involvement made to the approach or findings of the study.

Yes, the study has conducted scenario analyses to investigate the effect of engagement with LCS participants, through varying LCS uptake rates for the target population.

**Discussion**

**26. Study findings, limitations, generalizability, and current knowledge**

Report key findings, limitations, ethical, or equity considerations not captured and how these could impact patients, policy, or practice.

Yes, in the Discussion session, study key findings, limitations, generalizability, and current knowledge were reported.

**Other Relevant Information**

**27. Source of funding**

Describe how the study was funded and any role of the funder in the identification, design, conduct, and reporting of the analysis.

Yes, the study has described the funding and specified that the funder had no role in identification, design, conduct, and reporting of the analysis.

**28. Conflicts of interest**

Report authors’ conflicts of interest according to journal or International Committee of Medical Journal Editors requirements.

Yes, the study has reported the author's conflicts of interest.
